# Gender norms and access to sexual and reproductive health services among women in the Marrakech-Safi region of Morocco: a qualitative study

**DOI:** 10.1186/s12884-023-05724-0

**Published:** 2023-06-02

**Authors:** Hajar Ouahid, Adil Mansouri, Majda Sebbani, Nadia Nouari, Fatima Ezzahra Khachay, Mohamed Cherkaoui, Mohamed Amine, Latifa Adarmouch

**Affiliations:** 1 Bioscience and Health Research Laboratory, Faculty of Medicine and Pharmacy, Cadi Ayyad University, Marrakech, Morocco; 2 Clinical Research Department, Mohammed VI University Hospital, Marrakech, Morocco; 3 Department of Public Health, Community Medicine and Epidemiology, Faculty of Medicine and Pharmacy, Cadi Ayyad University, Marrakech, Morocco; 4 Laboratory of Pharmacology, Neurobiology, Anthropobiology, and Environment, Faculty of Sciences Semlalia, Cadi Ayyad University, Marrakech, Morocco

**Keywords:** Gender norms, Gender roles, Healthcare Access, Sexual health, Reproductive health, Morocco

## Abstract

**Introduction:**

Improving access to sexual and reproductive health remains a public health challenge, especially for women, whose access is affected by several determinants, such as gender inequality, which is the underlying barrier to all other determinants. Many actions have been carried out, but much remains to be done before all women and girls can exercise their rights. This study aimed to explore how gender norms influence access to sexual and reproductive health services.

**Method:**

A qualitative study was conducted from November 2021 to July 2022. The inclusion criteria were women and men aged over 18 years old, living in the urban and rural areas of the Marrakech-Safi region in Morocco. A purposive sampling method was used to select participants. Data were obtained through semi-structured interviews and focus groups with selected participants. The data were coded and classified using thematic content analysis.

**Results:**

The study highlighted inequitable, restrictive gender norms that lead to stigmatization and affect the sexual and reproductive healthcare-seeking behavior and access of girls and women in the Marrakech-Safi region. These most common gender norms for women include parental refusal, stigmatization, and social exclusion of girls from sexual and reproductive health education services; strong decision-making power of family members over contraceptive use and women’s adherence to pregnancy monitoring and access to supervised delivery; and culturally constructed role allocation, assigning a reproductive role to women and making them responsible for the health of new-borns.

**Conclusion:**

Sexual and reproductive health projects must strive to be gender sensitive. Gender-blind projects are missed opportunities to improve health outcomes and advance gender equality.

## Background

Sexual and reproductive health inequalities remain a significant challenge for women worldwide. Despite advances in healthcare and technology, women continue to face barriers to accessing critical services, information, and resources related to their sexual and reproductive health. These inequalities are particularly pronounced at different stages of a woman’s life, from adolescence to menopause and beyond [1, 2].

More than one in four women (27%) in low-income countries give birth before the age of 18. This represents approximately 12 million women in the least developed countries who gave birth during their adolescence [3]. Each year, an estimated 3.9 million girls aged 15–19 years undergo unsafe abortions [4]. Approximately 270 million women worldwide want but do not have access to contraception, and 830 women die every day from preventable causes related to pregnancy and childbirth [1, 5].

In Morocco, maternal mortality has decreased to 72.6 (2015–2016). However, this mortality remains higher in rural areas with a rate of 111.1 per 100,000 live births in rural areas versus 44.6 per 100,000 live births in urban areas [6]. Among the main causes of maternal death are unsafe abortions [7].

Access to sexual and reproductive health is affected by several determinants [8,9 ] such as gender inequality which is the underlying barrier to all other determinants, many actions have been carried out, but much remains to be done before all women and girls can exercise their rights [10].

In many countries, sexual and reproductive health services tend to target only married women and ignore the needs of adolescent girls and unmarried women because of social representations [11–13].

Gender norms are standards and expectations that women and men generally conform to, within a range that defines a particular society, culture, and community at a particular time. They are ideas about how women and men should be and act. Internalized early in life, gender norms can establish a lifelong cycle of socialization and gender stereotyping [14].

Gender norms contribute to social expectations that, in turn, lead to the control of sexuality and reproductive behavior for certain groups [15, 16]. It presents one of the most significant barriers to advancing the empowerment of women and girls to freely make decisions about their sexual and reproductive health and rights [17, 18]. It is a violation of human rights that affects all aspects of a woman’s life, and has many complex consequences for sexual and reproductive health [19, 20].

Gender is a determinant of health inequalities, both on its own and in combination (intersectionality phenomenon) with socioeconomic conditions, age, ethnicity, disability, sexual orientation, etc. [21]. Furthermore, gender affects all targets of Sustainable Development Goal 3, as it interacts with other determinants [22, 23].

Addressing gender norms, sexual and reproductive health inequalities requires a comprehensive approach that considers the different stages of a person’s life. A life course approach acknowledges that individuals experience health and social issues at different stages of their lives, and that these experiences are shaped by various factors, including gender norms, social determinants of health, and access to healthcare services. By taking a life course approach, interventions and policies can be developed that are more effective in promoting gender equality and improving sexual and reproductive health outcomes throughout an individual’s life. This approach requires a focus not only on individual behavior but also on broader societal factors, such as policies, cultural beliefs, and access to resources, which affect individuals across their lifespan [24].

Evidence suggests that incorporating a gender perspective into health programs leads to better outcomes. To enhance health outcomes and promote gender equality, all sexual and reproductive health initiatives should aim to be gender-sensitive. When developing, executing, and assessing a sexual and reproductive health program, prioritizing gender equality is crucial, particularly as gender disparity deprives women of their sexual and reproductive rights [25].

**Fig. 1:**
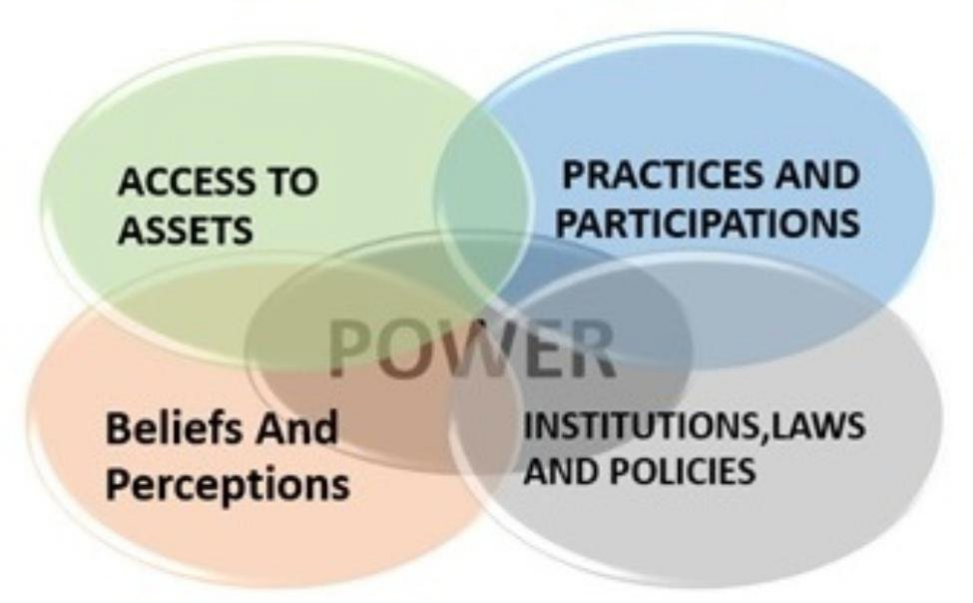
Gender Analysis Framework

In order to integrate this gender approach, it is important to conduct an analysis of the different determinants that surround it. For this purpose, a gender analysis framework was developed, which provides a structure for organizing information on gender roles and relations (Fig. 1) [26].

This framework allows us to systematize information about gender differences in different domains of social life and to examine how these differences affect people’s lives and health.

Research on the influence of gender norms on sexual and reproductive health is lacking, and no research has simultaneously addressed all the above issues and their impact on women’s health in Morocco, hence the importance of undertaking research on this issue to analyze this determinant in the Moroccan context. For this purpose, this study aimed to explore which gender norms influence access to sexual and reproductive health services in the Moroccan context.

## Method

### Study design

An exploratory qualitative study was conducted from November 2021 to July 2022. The target population were sampled with maximum variation in the urban-rural area of the Marrakech-Safi region.

### Study setting

The Marrakech-Safi region has a population of 4,520,569 (RGPH2 2014), a density of 115 inhabitants per km2, and corresponds to 5.5% of the national territory. The region has eight provinces and one prefecture. The number of communes is 215, of which 18 are urban and 197 are rural. (About 14% of all communes nationwide). The distribution of the urban and rural municipal population is 1,928,525 and 2,576,242 respectively [27]. We conducted interviews in not only the urban provinces and the prefecture of the Marrakech-Safi region, but also in rural areas that were selected based on their geographical accessibility, high population density, and proximity to health structures in the region. Additionally, focus groups were held in the province of Youssoufia, enabling us to gather participants from both the city and the surrounding rural areas.

### Participants and sampling

The inclusion criteria were women and men aged over 18 years old, living in the Marrakech-Safi region. To diversify perspectives, participants interviewed were from a variety of demographic backgrounds in terms of age, marital status, age at marriage, urban or rural residence, and education level. A purposive sampling method was used to select participants.

### Data collection

Data were obtained through semi-structured interviews and focus groups. These two focus groups, organized separately with one focus group for 10 women and another with 9 men, as well as 32 interviews, were conducted face-to-face, using the language commonly understood and spoken locally. Three authors of this study conducted the interviews. They ´were doctoral students at the epidemiology and public Health research laboratory. The sample included mothers- and fathers-in-law, community referents, single youth, and married people. Detailed field notes were taken to capture the language and nonverbal cues, as well as audio recordings. Barrier gestures against COVID-19 were observed during the sessions, such as wearing masks, maintaining social distance, and using hand sanitizer.

Interviews measures and focus groups with participants were conducted in private spaces which was chosen based on the participant’s preference and convenience, and that it was a location where they felt comfortable to speak.

We ensured that the location was secure and free from distractions or interruptions. The interviews and focus groups were recorded using a digital recorder with the consent of the interviewees and were then transcribed and compared with handwritten notes for the reliability of interpretation.

Each focus group session lasted an average of 53 min, and the length of the interviews was 45 min on average. Each participant consent was signed.

### Data collection tools

The interview guide was based on the gender analysis method. As defined by USAID, gender analysis is an analytical and social science tool used to identify, understand, and explain disparities between men and women within households, communities, and countries and the relevance of gender norms and power relations in a specific context.

It is a systematic method for examining differences in the roles and norms of women and men, girls, and boys; the different levels of power they hold; their different needs, constraints, and opportunities; and the impact of these differences on their lives and access to health services.

The four areas of the framework are:

- Access to assets: how gender relations affect access to the resources necessary for a person to be a productive member of society.
- Beliefs and perceptions: these stem from cultural norms or beliefs about what it means to be a man or woman in a specific society.
- Practice and participation: This dimension summarize information about the different roles of men and women, when and where they are active, their ability to participate in different types of economic, political, and social activities, and their decision-making.
- Institutions, laws, and policies: This dimension focuses on information about the formal and informal rights of men and women, and how they are affected differently by the policies and rules that govern institutions, including the health system. Power: permeates all domains and determines who owns, who can acquire, and who can use property, and who can make decisions about their bodies and children [26] .

### Data analysis

A thematic content analysis method was used to analyze the transcribed data. We began by transcribing the interview data verbatim. The interviews were conducted in Arabic dialect and were transcribed and translated to French by the authors, who are fluent in Arabic dialect as their mother tongue and also fluent in French and English. The translated transcripts were then analyzed in French by the research team. During the elaboration of this manuscript in english, the authors translated the verbatim from Arabic dialect to English.

Our data were coded around specific questions. Then We then undertook an active reading of the transcribed data, looking for meanings and identifying relevant themes. Thus, triangulation of information sources was used to improve the reliability of collected data, and the credibility of research findings was achieved through study authors verification and participants debriefing.

### Ethical considerations

Principles of research ethics were followed, including participant anonymity and informed consent. Informed consent was explained to the participants and obtained before the interview began. An information sheet was given to the participant. The confidentiality of data was respected. In addition, participants were free to participate and withdraw at any time. The University Hospital Ethics Committee of Marrakech has approved the study. The approval number is 112,022.

## Results

There were 51 participants in this study, 29 women and 22 men. The average age of participants was 37.7 ± 15.1 years old, their education levels varied from primary in 15.7% to university in 39.2% of them, while 23.5% were illiterate. Among these participants, 51% were married and 35.3% were single. Regarding their place of residence, 43.1% of the participants lived in rural areas, 51% of them already had children and 49% were employed (Table 1).

**Table 1:** Sociodemographic characteristics of participants

Variable		Number of participants (N = 51)	Percentage (%)
Gender	Female	29	56,9%
	Male	22	43,1%
Education level	Primary	8	15,7%
	Secondary	11	21,6%
	University	20	39,2%
	Illiterate	12	23,5%
Civil status	Single	18	35,3%
	Married	26	51%
	Divorced	4	7,8%
	Widowed	3	5,9%
Place of residence	Urban	29	56,9%
	Rural	22	43,1%
Participants with children		26	51%
Participants with a job		25	49%
Religion	Muslim	49	96,1%
	Jewish	2	3,9%

This study identified gender norms related to women’s sexual and reproductive health in the Marrakech-Safi region. The thematic content analysis highlighted seven themes defined in four categories and six sub-themes (Table 2).

**Table 2:** Identified Themes and sub-themes

Catégories	Thèmes	Sous-thèmes
**Access to resources and gender relations**	• Mobility Restrictions • Education and access to information	
**Roles, responsibilities and gender relations**	• Decision-making processes • Role assignment and gender relations	
**Beliefs and perceptions**	• Around Sexual Health • Gender and Access to Sexual and Reproductive Health Services	• Community Pressure and Contraception • Decision-making and Pre- and Post-natal Follow-up • Experience with the delivery process • Blame and rejection of people with STIs/AIDS • Involvement in newborn and child health • Menopause, the age of despair
**Institutions, Laws, and Policies**	• The social dilemma of unsafe abortions	

### Access to resources and gender relations

#### Mobility restrictions

Female participants claim to have some mobility restrictions outside of their homes, including not being allowed to leave the house without permission from their husbands for anything, such as being unable to go out for walks on their own, or look for a job. Also, girls do not have the same freedom to leave the house as the boys in the family.

((***If I get sick or one of my children gets sick, i don’t leave the house until my husband comes and he will decide if we will go to the hospital or not. I have no right to leave the house no matter what the situation is***)) (Female, 46 years old, rural municipality).

#### Education and access to information

Girls in some rural areas leave school right after primary school, a decision taken by parents to protect them from the risk of sexual harassment. ((***I dream of continuing my studies and get a work, but it is impossible, it is forbidden***)) (Female, 17 years old, rural municipality).

((***We were deprived from continuing our education in college because of our parents’ concern that if we attended secondary school, we could be able to do something dishonorable and harm the family’s reputation because we would be in a class with boys.***)) (Female, 33 years old, rural municipality).

### Roles, responsibilities, and gender relations

#### Decision-making process

Early marriage is still an issue in rural areas. ((***In general, girls in our douar get married at the age of 15 to 16, this is the right age for marriage, if the girl reaches for example 22 or 24 years is too much, she may not find a husband here***)) (Male, 43 years old, rural municipality).

Moreover, the women’s focus group emphasized the emotional harassment that women who delay getting married face from those around them. They consider them imperfect, no matter what their professional status and academic success she has achieved, marriage remains the guarantee of their position in society ((***At the age of 30, they called me a spinster, a nickname they attach to older single girls***)) (Female, 53 years old, Marrakech).

Particularly in rural places where the husband’s approval is required before a woman can go for a consultation or check her pregnancy, women are perceived to be less important than males. ((***After my husband agreed, i was allowed to have my antenatal appointments at the village health center.***)) (Female, 34 years old, rural municipality).

#### Role assignment and gender relations

The focus groups highlighted that society assigns a reproductive role to women and a productive role to men. This distribution of roles limits women’s access to tangible resources and economic independence. ((***I must take care of the girls, it is a task for women like household chores, and my husband must work and bring the money home***)) (Female, 24 years old, rural municipality).

The reproductive role represents the responsibility of reproduction and maintenance of the future workforce. It does not end with the birth of children but extends to include the responsibility of bearing and giving birth to children, providing for their care and upbringing, and domestic work. Despite the importance of this role, it is generally seen as unreal work, but part of women’s human nature, and this role seeks to limit women’s experiences to what is solely biological.

### Beliefs and perceptions

#### Around sexual health

Sexual health is a term that reflects a social taboo for participants who responded intimidated to our questions about sexuality. The same attitude was observed with focus group participants, who claimed that talking about sexuality could lead to suspicions of infidelity, especially for girls who asked about it, with a social prohibition on sexuality outside of marriage for young women, especially premarital sex. ((***In our culture, girls should not show their sexual needs and abilities, it is unacceptable! therefore, they hesitate and do not ask for care even when they need this kind of service, and relationships outside marriage are forbidden in our Muslim religion***)) (Male, 33 years old, Marrakech).

Some norms weigh on youth and contribute to limiting access to sexual health information, which is limited to information from social networks and websites. ((***I have never had any information on these subjects, it is shameful to talk about sexuality, and even when I got married, I was never able to discuss my sex life even with my husband, even with my relatives***.)) (Female, 26 years old, Safi).

Most parents do not give the necessary sexual education to teenagers since they have not received any training. Also, the fact that this subject represents shame and a lack of respect between people. The participants also revealed how difficult it was to discuss issues related to menstruation and genital growth even with their mothers, pointing out that the only thing that mattered was to preserve the honor of the family by remaining a virgin girl. ((***The girl’s hymen is sacrosanct to her, therefore keeping it intact till the wedding is crucial; otherwise, she runs the risk of facing severe repercussions from her family, including expulsion, execution, or physical abuse.)***) (Female, 23 years old, rural municipality).

#### Gender and Access to sexual and Reproductive Health Services

The focus groups also highlighted that men do not usually talk about issues related to reproductive health services and that they consider it a women’s issue since it is related to women’s and children’s health.

#### Community pressure and contraception

A barrier to accessing information and using contraception is the prejudice and stigma against girls in health services, and the lack of confidentiality for users who wish to learn about family planning issues. Unmarried girls are seen as dishonest prostitutes, and risk social isolation. Their presence at this facility is related to illegal sexual relations that are not accepted by society. ((***An unmarried woman does not have the right to seek information on contraceptives, it is a societal suicide***)) (Male, 33 years old, Marrakech).

Some participants highlighted religion as a factor that influences the use of contraception. ((***it’s Forbidden, it’s God who decides, children are a gift from God***)) (Male, 53 years old, Youssoufia).

Most of the male participants attributed the use of contraceptives by women as a role dedicated to them to avoid getting pregnant. ((***After I used the pill to space out my children, I wanted to put in the intrauterine device, but my husband didn’t agree, he told me that it will bother him in bed and that he might get hurt because of the IUD***)) (Female, 53 years old, Youssoufia).

Participants reported pressure from their partners and the broader community to prove a married couple’s fertility; they must have a child immediately after marriage and therefore women should not use contraception until they have their first child.

((***I endured harassment by my family because i had not had children yet, especially my father-in-law who verbally abused me every day claiming that i am a disgrace. At one point i started hitting my stomach, and i felt i handicapped.***)) (Female, 33 years old, rural municipality).

This pressure can push women towards practices that are risky for their health. ((***I was desperate, i had to get pregnant. Some friends advised me to go and see a women named ((FERAGA)), who inserted a herbal suppository into my vagina. I had a fever and got sick. These were the cause that led to an infection in my vagina***)) (Female, 53 years old, Marrakech).

On the other hand, participants added the importance of the sex of the newborn in their community. Having a male child was important for the value given to one’s social status and to ensure inheritance’s rights. Participants reported that the sex of the new-born was the main factor that pushed them to try to get pregnant as soon as possible to look for a boy. ((***I prayed to God to have a boy. Because in our community a woman must have a boy, others frown upon a man who does not have a male child in our douar***)) (Female, 24 years old, rural municipality).

Also, in the rural environment, families encourage having several children, especially males, so that they can contribute to the daily work. ((***I don’t want to get pregnant. I have three children, which is enough for me, but my husband insists on having more children***)) (Female, 33 years old, rural municipality).

#### Decision-making and pre-and post-natal follow-up

The focus groups claimed that it remains difficult for some women to take part in decisions related to referral to healthcare institutions in rural areas, other people can intervene in this decision making namely the husband, peers, and mothers-in-law. ((***I must always wait for my husband to take me to the health center to monitor my pregnancy; I was not allowed to go alone to consult***)) (Female, 33 years old, rural municipality). This difficulty is low among educated women with some financial independence, who in most cases are the main decision-makers regarding their health.

The influence of societal beliefs is present in pushing women not to continue their prenatal consultations and to be satisfied with a single consultation. ((***My husband doesn’t find any interest in going for more than one consultation, especially since the health center is far away in the village.***)) (Female, 24 years old, rural municipality).

The gender of the health care professional was a concern for some participants whose husbands prevented them from being examined by a male doctor. It’s a determinant that can influence the accessibility to supervised deliveries. ((***No, I do not agree, I prefer that she give birth at home than to be examined by male doctors. I do not understand why they accept them in these specialties that are supposed to be for women. It is disrespectful!***)) (Male, 53 years old, Youssoufia).

The involvement of men in the surveillance and monitoring of their partners’ pregnancies is low in rural areas and is increasingly marked in urban areas ((***My husband liked to accompany me to the gynecologist to see the little one in the ultrasound machine***)) (Female, 25 years old, Marrakech).

#### Experience with the delivery process

The experience of previous deliveries plays a very important role in the decision making of subsequent pregnancies and deliveries, some participants claimed bad experiences in healthcare institutions pushed them to try to give birth in their homes. Sexualized language and abuse of patients were mentioned in the discourses. ((***I had a bad experience giving birth at the hospital, it was a nightmare, especially some of the sentences that were addressed to me by the healthcare team, I did not feel comfortable, and they behaved badly toward me***)) (Female, 30 years old, rural municipality).

The lack of psychological support for women during childbirth, the non-involvement of the husband in the delivery process, as well as the underestimation of the effort made by women in the face of the pain of contractions and expulsion, influenced women’s health status. Some males participants attributed childbirth as a natural role of women and that they should assume the consequences. ((***It is nature, they must stop complaining, and God has favored them for the suffering they undergo***)) (Male, 49 years old, Marrakech). Others call for the importance of supporting women in this difficult phase in which some women feel alone and neglected.

#### Blame and rejection of people with STIs/AIDS

Gender stereotypes blame women for the spread of HIV/AIDS. Often, they are the first to be tested because they are pregnant, or because they have a sick child. They are the first to be judged.

Added to this is the stigma that accompanies such infections, which can prevent access to healthcare institutions, as well as the negative attitudes of some healthcare professionals toward infected women. ((***I once had vaginal pruritus that developed into a condyloma, so my husband had it first before me, so we didn’t go to see a doctor, since any disease of this kind is related to illegitimate sexual practices.***)) (Female, 33 years old, rural municipality).

Some participants lack information about sexually transmitted infections and the level of risk involved in unprotected sex. Despite being sexually active, talking about these types of infections could lead to prejudices that suspect those discussing these topics of being depraved people. ((***Only prostitutes and their partners can contract these diseases.***)) (Female, 53 years old, Youssoufia).

Also, participants pointed out the importance of highlighting high school courses that deal with this topic to students so that they can benefit from good awareness and learn about these health risks. ((***I instruct a course on this topic in my field, but because it is the last one of the year and my students have to take a regional exam, they do not pay much attention to it.***)) (Female, 43 years old, Youssoufia).

Some participants mentioned the importance of integrating parents for the sensitization on these infections. This comes from the fact that some students do not manage to communicate with their parents on these subjects, for fear of being punished or isolated. ((***After being suspected of having syphilis, the mother of one of my students preferred to treat him with herbs for fear of the reaction of the father and his entourage, unfortunately, his condition became complicated***)) (Female, 43 years old, Youssoufia).

People prefer to look for this kind of information on social networks in a discreet way to avoid the judgments of their relatives, but the shared information is not framed, based on scientific data, and can be misleading. ((***Most of the time i search on Google and we discuss this stuff anonymously on Facebook groups, but i’m not sure of the quality and veracity of the information***)) (Female, 34 years old, Marrakech).

#### Involvement in newborn and child health

The father frequently makes all of the health-related decisions for the kids. On the other hand, the people who take care of the children are mostly women ((***I am the one who takes care of my children, it is my responsibility, once i asked my husband to look after them, he answered me you are crazy you think i am a nanny for your children***)) (Female, 24 years old, rural municipality). It is a role assigned by default in this context.

Some messages addressed in health care institutions for health promotion purposes may be stereotypical, assigning roles to mothers during sensitization sessions while neglecting the involvement of husbands in this care. ((***At the hospital, he was explaining to us things related to breastfeeding and taking care of newborns, but it was only meant for women, I never saw a man accompanying his wife it will be a shame***)) (Female, 23 years old, rural municipality).

#### Menopause, the age of despair

Menopause is the object of many stereotypes. It is considered an end to the sexual life of women. It is still taboo. Menopause is often considered a marker of women’s old age and the loss of ovarian functions, which affects femininity and fertility ((***it’s over for me, I’m useless in fact***)) (Female, 68 years old, rural municipality).

However, the majority of the participants informed us that the emphasis is given more to women of reproductive age and that as soon as women pass the stage of menopause, they lose the value of their social position. The necessity for health is always present for the prevention of gynecological malignancies and even for sexual health. ((***It is a fatal stage, I suffer from a lot of disorders, hot flashes, sweats, and pains in my knees kill me, but I prefer not to say anything to my husband***)) (Female, 48 years old, Marrakech).

### Institutions, laws, and policies 

#### The social dilemma of unsafe abortions

Most of the participants agreed that the main cause that will push an unmarried woman to carry out an abortion is the rejection of the whole community, and even the risk of being physically attacked by her entourage. ((***It is the worst shame, she absolutely must have an abortion to hide her scandal, if her family finds out, they may kill her. The honor of the whole family will be at stake***)) (Female, 46 years old, rural municipality).

Children resulting from relationships outside of marriage are considered illegitimate children and will be deprived of inheritance and succession rights. ((***I see no other solution for an unmarried pregnant woman than to abort her child since in our country the law does not protect her child and she will have problems both socially and legally***)) (Female, 43 years old, Youssoufia).

Most single women find themselves alone to deal with this problem, hence the fact that some of them turn to clandestine methods to end their pregnancy before it is visible to society. ((***I was still young, and i was afraid, my father could kill me, finally, my sister brought me a mixture of herbs bought at the herbalists in the souk, it was difficult but fortunately i ended up having an abortion, i bled a lot***)) (Female, 22 years old, Marrakech). On the other hand, male participants reported the pressure of society on this issue and that they feel obliged to push their partners to have an abortion.

Regarding the case of married women, clandestine abortions can be the recourse in front of unintentional pregnancies, the participants affirmed to us that the socioeconomic level is the principal factor that can push women to carry out an abortion. ((***I had 5 children, and the last one was only 2 months old; it was a mistake, i went with my husband to a gynecologist who gave me an injection to stop the pregnancy. If i kept this pregnancy everyone would call me a big rabbit***)) (Female, 55 years old, Youssofia).

## Discussion

This study discovered that gender norms affect how girls and women in the Marrakech-Safi region seek out and access sexual and reproductive health care due to cultural and societal norms, of which social stigma is particularly prevalent.

The results of this study indicate that misconceptions about sexual health needs lead to the stigmatization and social exclusion of young girls from sexual health education services. Some adults, including parents, believe that providing information about sexuality would lead their daughters to engage in sexual activity, which is unacceptable in their communities. Although several studies have shown this to be incorrect, Kirby et al. conducted a review of 83 studies measuring the impact of sex and HIV education programs on sexual behavior among young people under age of 25 worldwide and concluded that this type of sex education does not encourage sexual behavior [28]. Thus, there is strong evidence that programs do not accelerate or increase sexual behavior, but rather that some programs delay or decrease sexual behavior and contribute to increased condom or contraceptive use [29].

Data from our study showed that open conversation about sexual health is still taboo and that parents do not feel comfortable discussing sexual and reproductive health topics with their daughters. This translates into their lack of knowledge about sexual health issues and especially about where to get information and services. As a result, young girls are unable to access the knowledge and skills needed to make healthy decisions. This is consistent with other research that highlights the lack of sexual health training for nurses working to care for SRH service users [30]. Also, results from other studies have shown that participants identified the need to reduce stigma, standardize sexual health care, improve convenience, and affordability, ensure confidentiality, and increase awareness of services as essential to improve the access [31, 32].

On the other hand, the results of this study highlighted the gender norms that influence access to different contraceptive methods for women in this context, the need for which should be met only for married women. These gender norms contribute to limiting the access of young unmarried women to these services, calling them easy girls, and dishonest, and that their presence even to get information on this subject will be a “societal suicide”. Similar findings have been demonstrated by other researchers in South Africa, whose studies highlighted difficulties in accessing SRH services, including non-supportive attitudes of providers, unequal power dynamics in relationships, and communication problems with parents and community members [33, 34].

Moreover, our data also showed that married women also have societal constraints influencing their access to and use of contraceptives, namely the power of peers and the community, which prevent them from using any contraceptive method until they have fulfilled their reproductive role of having several children, especially male ones, without respecting the spacing between pregnancies. The same observation has been made in previous studies conducted in Africa, which found that the approval of husbands is a determining factor in the adoption of modern contraception by their wives [35, 36]. This situation is strongly present in rural areas and is explained by the strong decision-making power of men who have higher fertility preferences than women. This has been explored by other studies which have argued that gender norms, limited autonomy, and lack of decision-making power limit women’s ability to access maternal healthcare services [37].

Our study found inequity in the choice of contraceptive methods used to space births. This choice may be influenced by several factors, including beliefs about the negative effects of contraceptive methods and the husband’s refusal. The woman may be unable to negotiate condom use with her partner. Especially since many husbands did not understand family planning well because they perceived it as a woman’s issue. Other studies have highlighted that gender norms in fertility and birth planning issues were also considered to be a woman’s concern [38]. Thus, engaging men in communication about family planning was seen as inappropriate and distracting, including in awareness campaigns, which have traditionally targeted women [39, 40].

Although women’s adherence to pregnancy monitoring and access to assisted delivery is relative to the economic status of the household, it is also influenced by gender norms, namely the refusal of some husbands of male providers, and women’s financial and decision-making autonomy to be able to navigate antenatal care and assisted delivery without waiting for a third party’s approval. In addition to this, the results of this study showed that previous birth experience plays a major role in the orientation of the subsequent birth. The behavior of health care personnel during childbirth is a determinant of this experience, some of which may address gendered remarks that affect the physical and psychological integrity of parturients, something that has been described as emotional and gendered harassment by some participants in this study, and subsequently generate a refusal to use these care facilities and risk home births.

This topic was the focus of several studies, Azad et al. showed that internal and external gender discrimination contributes to women’s disproportionate lack of access to care. As well as economic empowerment alone is not enough to correct these socially constructed roles, and they found that education is a protective factor against gender disparities [41]. However, Morganet al. have argued that failure to address underlying gender dynamics limits the sustainability of SRH progress. Gender power relations can be understood in terms of how power is constituted and negotiated concerning access to resources, division of labor, social norms, and decision-making, the intersection of which has affected access to and utilization of maternal health care in Uganda [42].

In terms of husbands’ involvement in newborn health and analysis of culturally assigned gender roles, our study demonstrated the influence of mothers-in-law and generational power dynamics. Women perform almost all tasks related to newborn care on their own, while men assume the traditional responsibilities of economic providers and decision-makers, especially regarding the health of their wives and children. These findings have also been reported in previous studies that have shown that in the family setting “caring” for the newborn also follows gender and generational logic, which shows a better integration of medical advice and prescriptions by young women. Also, in health centers, the people who play the role of accompanying persons are generally women [43, 44].

Illegitimate abortion remains a taboo subject in community discussions and a public health issue, especially given the risks associated with this type of practice. Our study identified the factors that push women to initiate an illegitimate abortion as being the rejection of the whole community and the physical and psychological violence from family members. However, this practice is prohibited in the Muslim religion. In addition to these factors, the social status of childrens from an extramarital relationship is considered illegitimate and these children will be deprived of inheritance and succession rights according to the Moroccan constitution (Article 32) [45] and the Moroccan family code (Article 148) [46]. This issue is at the heart of international discussions in different countries. In the United States, Abigail S. Cutler et al. identified factors associated with high stigma and less favorable views toward abortion care policies as being of Catholic, evangelical, or Protestant religion and Republican political affiliation [47]. On the other hand, the results of a study in China highlight the problem of repeated abortions and suggest the need for the government and civil society to intensify their efforts to reduce the risk of unwanted pregnancies and repeated abortions in China. This can be achieved by improving access to knowledge about reproductive health and contraception for women and their sexual partners and by promoting their correct, consistent, and effective contraceptive practices [48].

Inequitable and restrictive gender norms lead to the stigma that can prevent the use of STI/HIV services. The results highlighted gender-related difficulties such as inaccessibility to correct information about sexually transmitted infections and the risks of unprotected sex. Also, the social prejudices that consider people discussing these issues as an immoral person, and some behaviors of health professionals that do not give enough confidence to the subjects with these infections to confide and access care and follow-up services. Several research studies have addressed this issue, such as Leddy et al., conducted their study in a community mobilization intervention in rural South Africa and aimed to change gender norms to improve the utilization of HIV services. Their results showed that norms about men’s reluctance to seek help, men’s expected control over women, and the difficult norms that women are solely responsible for the health of the family need to be challenged [49, 50].

### Strengths and limitations

Our study has some limitations namely, the use of self-reported data. Respondents may have responded in a way that they believed to be socially acceptable for the research data and the researcher, which could lead to a lack of some other details about gender disparities. Also, the results of the present study may not be generalizable to the general population and other different socio-cultural, economic, and geographic contexts. On the other hand, the recruitment of participants was voluntary, so potential participants who prefer not to participate may have different important opinions from those who agreed to participate.

Another potential limitation is the loss of information when translating the transcripts from dialect to French. Therefore, to control this limit, interviewers who are fluent in Arabic dialect as their mother tongue and fluent in French and English did the translation, and a second check was made for consistency.

On the other hand, our study has some important strengths. We included multiple groups of participants in this study from a variety of demographic backgrounds in terms of age, age at marriage, urban or rural residence, and education level to diversify perspectives and capture a range of relevant viewpoints, thereby increasing the richness of the data.

In addition, recruiting participants from different urban and rural areas allowed us to capture broader and contextually relevant data affecting access to and use of sexual and reproductive health services. In addition, we applied different data collection methods to achieve data saturation and performed triangulation to improve the credibility of the results.

### Perspectives and recommendations

To carry out advocacy based on the evidence of our study, we recommend that policy makers should integrate a gender approach into the development of public health policies. This can be achieved by considering the following:

- Inclusion of gender-disaggregated data in health research to identify gender differences and to develop policies that address gender-specific needs.
- Training of healthcare providers on gender sensitivity to ensure equitable access to healthcare services.
- Implementation of gender-sensitive health policies that take into account the different health needs of men and women.
- The implementation of sexual and reproductive health programs in schools and universities is strongly recommended.
- Health care professionals should incorporate a gender approach into their professional practice to provide quality care.
- Additional research should be conducted to assess the level of knowledge of gender norms among health professionals.

Furthermore, it is important to consider the cultural context in which these policies are developed and implemented. For example, in our study, we found that women faced significant barriers to accessing healthcare due to cultural norms that prioritize the health needs of men over women. Policy makers should therefore work with local communities to address these cultural norms and ensure that healthcare services are accessible and equitable for all.

## Conclusion

This study explored important barriers to accessing sexual and reproductive health services that adhere to more traditional gender norms. Failure to address the underlying dynamics of these norms affects the consolidation of gains in the fight against maternal mortality, prevention and treatment of sexually transmitted infections and unwanted pregnancies, and other sexual and reproductive health issues.

Sexual and reproductive health projects must strive to be gender sensitive. Gender-neutral projects are missed opportunities to improve outcomes in access to these care services and advance gender equality.

## Data Availability

The data supporting the conclusions of this article are not publicly available because further research remains ongoing but are available from the corresponding author on reasonable request.
